# Independent Manipulating of Orthogonal-Polarization Terahertz Waves Using A Reconfigurable Graphene-Based Metasurface

**DOI:** 10.3390/ma11101817

**Published:** 2018-09-25

**Authors:** Li Deng, Yuanyuan Zhang, Jianfeng Zhu, Meijun Qu, Ling Wang, Chen Zhang

**Affiliations:** 1 Beijing Key Laboratory of Network System Architecture and Convergence, Beijing University of Posts and Telecommunications, No.10 Xitucheng Rd., Beijing 100876, China; zhujianfeng@bupt.edu.cn (J.Z.); qumeijun@bupt.edu.cn (M.Q.); lingwang@bupt.edu.cn (L.W.); zhangchenzc@bupt.edu.cn (C.Z.); 2 School of Information and Communication Engineering, Beijing University of Posts and Telecommunications, No.10 Xitucheng Rd., Beijing 100876, China; zhangyuanyuan@bupt.edu.cn

**Keywords:** graphene, metasurface, reconfigurable, orthogonal-polarization

## Abstract

Viewing the trend of miniaturization and integration in modern electronic device design, a reconfigurable multi-functional graphene-based metasurface is proposed in this paper. By virtue of the reconfigurability of reflection patterns, this metasurface is able to independently manipulate orthogonal linearly polarized terahertz wave. The building blocks of the proposed metasurface are series of graphene-strips-based unit-cells. Each unit-cell consists of two orthogonal graphene strips and a grounded substrate, which has anisotropic responses for each of orthogonal polarizations (x-polarized and y-polarized waves). The reflection phases of both x- and y-polarized waves can be controlled independently through separate electrical tuning. Based on the proposed metasurface, functionalities including beam splitting, beam deflecting, and linear-to-circular polarization converting using a shared aperture are numerically demonstrated and analyzed. Simulation results demonstrate excellent performance, which is consistent with the theorized expectations. This work paves the way for enhancing the miniaturization of modern electronic/optical devices and potentially has important applications in the next-generation information systems for communication, sensing, and imaging.

## 1. Introduction

Metasurface, a two-dimensional (2D) equivalence of metamaterial, has been proposed as an effective way to arbitrarily manipulate amplitude, phase, and polarization of electromagnetic (EM) waves. Recently, many novel metasurface-based devices [1] have been proposed and analysed, such as emitters [2], absorbers [3,4,5], anomalous reflection or refraction metasurfaces [6,7,8], focusing metasurfaces [9], polarization beam splitters [10], cross-polarization converters [11], polarization rotators [12,13] and vortex EM wave generators [14], etc. Afterwards, these metasurface-based devices have been widely used in various applications such as sensing [15,16], communication [17], imaging [18] and cloaking [19], according to their remarkable wave manipulating capability on the sub-wavelength scale, low loss, and easy on-chip integration. Most previously proposed mono-functional metasurfaces, however, hinder the development of miniaturization and integration. Viewing the huge sizes and energy consuming resulted from mono-functional metasurfaces, there is a considerable demand for realizing metasurfaces with multiple functions, so as to improve the degree of integration of modern electronic or optical systems. Therefore, integrating multi-function into a shared metasurface is a meaningful work.

Recently, graphene, a monolayer of carbon atoms, arranged in a honeycomb lattice [20], has drawn substantial attention from the scientific community. Through chemical doping or electrical gating [21], the Fermi level and complex surface conductivity of graphene can be effectively tuned, exhibiting its advantage over conventional metals. Thanks to its unique electronic properties and noticeable electrically tunning capability, several functional graphene-based metasurfaces have been conceptually reported and demonstrated [22,23,24,25,26,27]. However, most of these works on graphene-based metasurfaces focused on fixed metasurfaces, which is not capable of reconfiguration. For example, Ref. [23] presents a graphene metasurface-based reflect array at terahertz band using different widths of the graphene patches to obtain different reflected phase on the surface. Ref. [24] efficiently realize anomalous reflection, reflective focusing lenses, and non-diffracting Airy beams based on graphene-based metasurfaces, but it requires three different array distributions to realize corresponding functionalities. In Ref. [25], although demonstrating a flat dual-band focusing reflector by using stacked graphene ribbons, it also depends on engineering the plasmonic resonance in graphene ribbons with different widths to obtain specific surface phase distributions, and the functionalities are fixed. In these works, only mono-functional reconfigurability in frequencies, polarizations [26] or reflected/transmitted patterns [27] is investigated while integrating more functionalities into shared apertures of metasurfaces are barely taken into account. To the best of our knowledge, multi-functional reconfigurable graphene-based metasurface with a shared aperture which only depends on electrical tuning was rarely reported in previous literature, in spite of the great demand.

In this paper, a reconfigurable multi-functional graphene-based metasurface is proposed, for the effect of greatly increasing the degree of miniaturization and integration of modern electronic devices. By virtue of the reconfigurability of reflected patterns, the proposed graphene-based metasurface can independently manipulate orthogonal linearly polarized terahertz waves. The building blocks of the proposed metasurface are series of graphene-strips-based unit-cells. Each unit-cell consists of two orthogonal graphene strips and a grounded substrate, which has anisotropic responses for each of orthogonal polarizations (x-polarized and y-polarized waves). The normal incident waves can be entirely reflected by the metal-grounded plane on the bottom of the metasurface and the reflection phases of both x- and y-polarized waves are controlled independently via separate electrical tuning. Based on the proposed design, four kinds of functionalities using a shared aperture are numerically demonstrated and analyzed for polarization beam splitting, beam deflection, and linear-to-circular polarization conversion with a deflection angle.

## 2. Properties of Graphene-Based Unit-Cell

**Figure 1:**
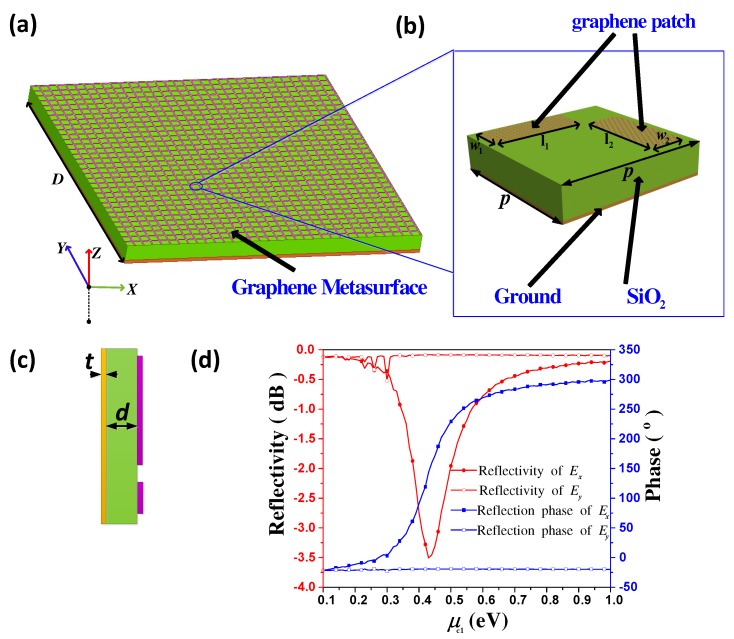
(**a**) The schematic of graphene-based metasurface, which can be generated by extending unit-cell along both of *x* and *y* directions; (**b**) The graphene-based unit-cell, which is composed of two top layer graphene strips and a grounded quartz glasses (SiO${}_{2}$) substrate; (**c**)The side view of the unit-cell; (**d**) The simulated reflectivity and reflection phase at 1.7 THz for both of x- and y-polarized normally incident waves with fixed $\mu_{c2}$ and varied $\mu_{c1}$ from 0.1 eV to 1 eV.

Electromagnetic waves in THz frequencies can excite prominent plasmonic resonances in graphene. But for the purpose of practical application, these wave-graphene interactions need to be further enhanced. Therefore, using the Fabry–Perot resonant principle [28], we design a unit-cell structure which can greatly enhance the wave-graphene interactions. The proposed Fabry–Perot resonant-based unit-cell consists of two orthogonal graphene strips and a square grounded quartz glasses (SiO${}_{2}$) substrate. When illuminated by THz waves, plasmonic resonances can be excited in the top layer graphene strips. The bottom metallic ground is designed for totally reflecting the incident waves. The proposed metasurface is generated by periodic extending the graphene-based unit-cells along both of the *x* and *y* directions, as shown in Figure 1a. It is worth noting that, in order to realize independent controlling of x- and y-polarized waves, two graphene strips must be perpendicularly placed, as demonstrated in Figure 1b. Here, the reason why we choose two orthogonal graphene strip structure is that it is maybe the simplest structure which can maximally decrease the mutual coupling of the two orthogonal polarizations, compare with many complex structures. The optimal dimensions of the unit-cell shown in Figure 1b are *p* = 14 μm, $w_{1}=w_{2}$ = 2 μm and $l_{1}=l_{2}$ = 10 μm. Similar to our previous work [29], parameters $l_{1}$ and $l_{2}$ can be changed independently to determine the reflection phases of x- and y-polarized waves, respectively, with fixed optimal parameters $w_{1}$ and $w_{2}$. Thus, $l_{1}$ and $l_{2}$ are two crucial parameters in this unit-cell to control the reflection phase of the incident wave. Obviously, in this unit-cell structure, x-polarized incident wave only sensitive to the plasmonic resonance in the *x* direction, corresponding to the variation of parameter $l_{1}$. And in contrast, y-polarized incident wave only sensitive to the plasmonic resonance in the *y* direction, corresponding to the variation of parameter $l_{2}$. Therefore, unit-cell designed by this geometrical changing method is obviously an element of fixed metasurface. Once this metasurface is fabricated, it cannot be changed. For achieving a reconfigurable metasurface, we must find a dynamic method to manipulate the unit-cell. In principle, controlling reflection phase by changing parameters of $l_{1}$ and $l_{2}$ is essentially independent changing the impedance of the x- and *y* direction. Thus, considering the impedance controlling capability of graphene through chemical potential tunning, we can equivalently change these geometrical parameters by tunning the chemical potential of the graphene strips. Concretely, we use chemical potential $\mu_{c1}$ to control strip 1, and chemical potential $\mu_{c2}$ to control strip 2, respectively. Then, changing of $\mu_{c1}$ means an equivalent changing of $l_{1}$, and similarly, $\mu_{c2}$ is corresponded to $l_{2}$, respectively. With this approach, a fixed unit-cell has changed to a reconfigurable unit-cell, and we can easily realize it through practical technologies such as chemical doping or electrical gating.

In order to obtain better understanding of the proposed unit-cell, detailed analytical methods such as multi-layer [30], transmission line theory [31], or effective medium theory [32] are always inevitable. But, in this paper, the total effective conductivity of the air-unit-cell interface which consists of two orthogonal graphene strips is difficult to calculate in an analytical way, although the conductivity of graphene strip can be easily determined by Drude model. Therefore, for verifying the performances of the proposed unit-cell, numerical simulation based on Ansoft HFSS 2017 software is applied. Figure 1c exhibits the side view of the proposed unit-cell model, where parameters *t* and *d* represent the thickness of SiO${}_{2}$ substrate and the bottom metallic ground plane, respectively. In our simulation, the relative permittivity of SiO${}_{2}$ substrate is $\epsilon_{r}=3.75$, and its loss tangent is tanδ=0.0184 [23]. The thickness of the substrate and the ground are set to *d* = 25 μm and *t* = 10 nm, respectively. The bottom metallic material is selected as Au. Importantly, we use the impedance boundary condition of HFSS to characterize the graphene strip, and the surface impedance of the graphene strip is represented as $Z_{s}=1/\sigma$. σ is the complex surface conductivity of the graphene strip, which can be approximated by the Drude model [33]

(1) $$\sigma =\frac{q_{e}^{2}k_{B}T\tau }{\pi \hbar^{2}\left(1+j\omega \tau \right)}\times \left[\frac{\mu_{c}}{k_{B}T}+2\ln\left(e^{-\frac{\mu_{c}}{k_{B}T}}+1\right)\right]$$

where $q_{e}$ denotes the elementary charge, $k_{B}$ is the Boltzmann’s constant, *ℏ* is the reduced Planck’s constant. T is temperature, τ is the relaxation time, ω is the radian frequency, and $\mu_{c}$ is chemical potential. It is worth noting that the relaxation time τ is a function of the carrier mobility μ, the Fermi energy $E_{f}$, and the Fermi velocity $v_{f}$. It can be calculated as $\tau =E_{f}\mu /ev_{f}^{2}$. In this work, the frequency is chosen to 1.7 THz, the room temperature is set to 300 K, the typical value of the carrier mobility μ is set to 10,000 cm${}^{2}$/Vs, the Fermi velocity $v_{f}=10^{6}$ m/s, and the Fermi energy $E_{f}$ is modeling as a variable corresponding to the chemical potential. Figure 1d demonstrates the simulated reflectivity and reflection phase at 1.7 THz for both of x- and y-polarized normally incident waves with fixed $\mu_{c2}$ and varied $\mu_{c1}$ from 0.1 eV to 1 eV. It is demonstrated acceptable x-polarized wave reflectivity above −3.5 dB and reflection phases varied from −20${}^{\circ }$ to 300${}^{\circ }$ (a phase span of 320${}^{\circ }$). Simultaneously, the y-polarized reflectivity and phases are almost kept in constant. It means that tunning $\mu_{c1}$ will affect the reflection properties of the x-polarized wave while keeping the y-polarized wave unchanged. Similarly, according to the symmetrical structure of the proposed unit-cell, $\mu_{c2}$ will only affect the reflection properties of the y-polarized wave. Therefore, we conclude that the reflectivity and reflection phases of x- and y-polarized waves can be manipulated independently by tuning parameters of $\mu_{c1}$ and $\mu_{c2}$, respectively. Meanwhile, a wide reflection phase span of 320${}^{\circ }$ can be achieved through our design. Importantly, a phase span of 270${}^{\circ }$ is sufficient to provide good performance in practical applications [34]. Furthermore, we can achieve reflectivity larger than −3.5 dB, enabling the design of multi-functional graphene-based metasurfaces to control reflected THz waves with acceptable efficiency. It is worth noting that the plasmonic resonance of metals becomes less pronounced as operating in THz frequencies because of the weaker interaction between electrons and waves. Therefore, the metallic reflection structures operating in THz frequencies often have a reflectivity less than 30% [35,36], which obviously have lower efficiency than our proposed graphene-based structure.

## 3. Design and Numerical Results of Proposed Reconfigurable Metasurface

**Figure 2:**
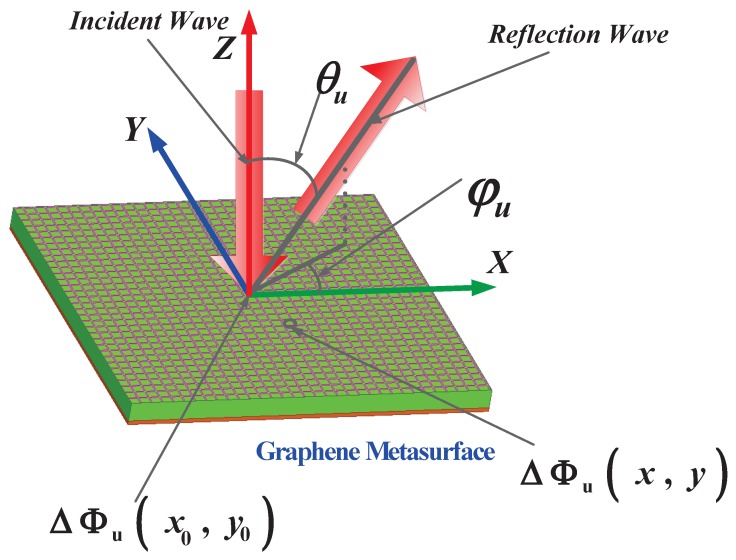
Illustration of wave deflection and definition of angles, according to Equation (2). *u* represents x- or y-polarization, ($\varphi_{u}$, $\theta_{u}$) represents the deflection direction of x- or y-polarized waves, $\Delta \Phi_{u}\left(x,y\right)$ denotes the reflection phase in position (x,y), and $\Delta \Phi_{u}\left(x_{0},y_{0}\right)$ denotes the reflection phase in the reference position $\left(x_{0},y_{0}\right)$. $\varphi_{u}$ is the angle between the projection of x- or y-polarized wave in the XOY plane and the +x axial, and $\theta_{u}$ is the angle between the direction of the wave and the +z axial.

Based on the proposed reconfigurable unit-cell of graphene-based metasurface, we can realize multi-functional applications. Here, four functions are integrated into a shared metasurface aperture. For convenience of expression, F1, F2, F3, and F4 are named for these four different functions, respectively. F1 and F2 are the two similar functions of beam splitting, F3 is the function of beam deflecting, and F4 is the function of linear-to-circular polarization converting, respectively. All these four functions are realized by using a shared aperture with different reflection phase distributions. Phase distributions can be calculated by generalized Snell laws of reflection [24].

(2) $$\Delta \Phi_{u}\left(x,y\right)=\Delta \Phi_{u}\left(x_{0},y_{0}\right)-\frac{2\pi }{\lambda_{0}}sin\theta_{u}\left[xcos\left(\varphi_{u}\right)+ysin\left(\varphi_{u}\right)\right]$$

where *u* represents $E_{x}$ or $E_{y}$, $\lambda_{0}$ is the wavelength in free space, ($\varphi_{E_{x}}$,$\theta_{E_{x}}$) and ($\varphi_{E_{y}}$,$\theta_{E_{y}}$) represent the deflection directions of x- and y-polarized waves, and $\Delta \Phi_{u}\left(x_{0},y_{0}\right)$ denotes an arbitrary reference reflection phase in position $\left(x_{0},y_{0}\right)$. Illustration of wave deflection and definition of angles are shown in Figure 2. Then, these reflection phase distributions of the x- and y-polarized waves can be tuned through $\mu_{c1}$ and $\mu_{c2}$, respectively. Thus, we can independently tune the deflection angles of x- and y-polarized waves according to Equation (2). It is worth noting here that the $\theta_{E_{x}}$ or $\theta_{E_{y}}$ is defined as the angle between the directions of x- or y-polarized wave and the +*z* axial, respectively. The coverage of θ is (−90${}^{\circ }$,90${}^{\circ }$), negative angle means anticlockwise rotation from the +*z* axial. Similar, the $\varphi_{E_{x}}$ or $\varphi_{E_{y}}$ is defined as the angle between the projection of the x- or y-polarized wave in the XOY plane and the +*x* axial, respectively, and the coverage of φ is (0${}^{\circ }$,360${}^{\circ }$). In our simulations, we apply a normally illuminated plane wave as an excitation at 1.7 THz. For avoiding confusing, we use uppercase ΔΦ to represent the reflection phase, and lowercase Greek letter φ to represent the deflection azimuth angle in this paper.

### 3.1. Graphene-Based Metasurface for Beam Splitting

**Figure 3:**
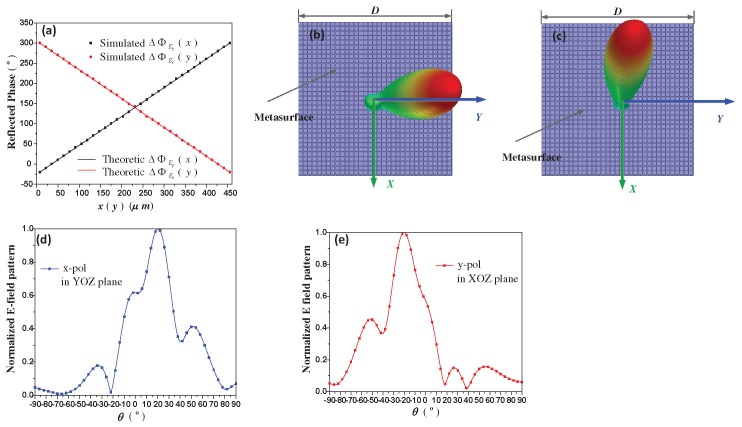
(**a**) The required phase distribution of function F1. $\Delta \Phi_{E_{x}}\left(y\right)$ represents the x- polarization wave reflection phase distribution along the +y direction, which is an one-dimension (1D) form of $\Delta \Phi_{E_{x}}\left(x,y\right)$ when *x* keeps constant in Equation (2). Similarly, $\Delta \Phi_{E_{y}}\left(x\right)$ represents the y-polarization wave reflection phase distribution along the +x direction, which is an one-dimension (1D) form of $\Delta \Phi_{E_{y}}\left(x,y\right)$ when *y* keeps constant in Equation (2); (**b**) and (**c**) are the 3-D scattering patterns of x- and y-polarized waves, respectively. Theoretic results are obtained from analytical calculation, and simulation results are obtained from numerical simulation, respectively; (**d**) and (**e**) are the 2-D scattering patterns of x- and y-polarized waves, respectively.

Figure 3 exhibits simulation results of the function F1. F1 aims to deflect the x- and y-waves to the directions of ($\varphi_{E_{x}}$ = 90${}^{\circ }$, $\theta_{E_{x}}$ = 20${}^{\circ }$) and ($\varphi_{E_{y}}$ = 180${}^{\circ }$, $\theta_{E_{y}}$ = −20${}^{\circ }$), respectively. According to Equation (2), reflection phase distribution is theoretic calculated as in Figure 3a, where the required reflection phase of x-polarized wave is gradually decreased along the +y direction, and similarly, the reflection phase of y-polarized wave is gradually increased along the +x direction, with a constant phase gradient. We can determine the exact value of chemical potential in each unit-cell by the relationship of reflection phases and chemical potentials provided by Figure 1d. Figure 3a also demonstrates 33 discrete reflection phases extracted from Figure 1d according to the phase distribution requirement. In this case, $\mu_{c1}$ is gradually changing along the +y direction, while keeping constant along the +x direction. Contrarily, $\mu_{c2}$ is gradually changing along the +x direction, while keeping constant along the +y direction. Figure 3b,c show the 3-D scattering patterns of x- and y-polarized waves, respectively. And 2D scattering patterns in Figure 3d,e are provided for more clearly presenting the beam deflecting effects. It is clearly demonstrated that the x- and y-polarized waves are deflected to the expected directions of ($\varphi_{E_{x}}$ = 90${}^{\circ }$, $\theta_{E_{x}}$ = 20${}^{\circ }$) and ($\varphi_{E_{y}}$ = 180${}^{\circ }$, $\theta_{E_{y}}$ = −20${}^{\circ }$), respectively. It means that we have realized a graphene-based metasurface which can split the x- and y-polarized components of arbitrarily linear polarized incident wave to a specific direction, respectively.

**Figure 4:**
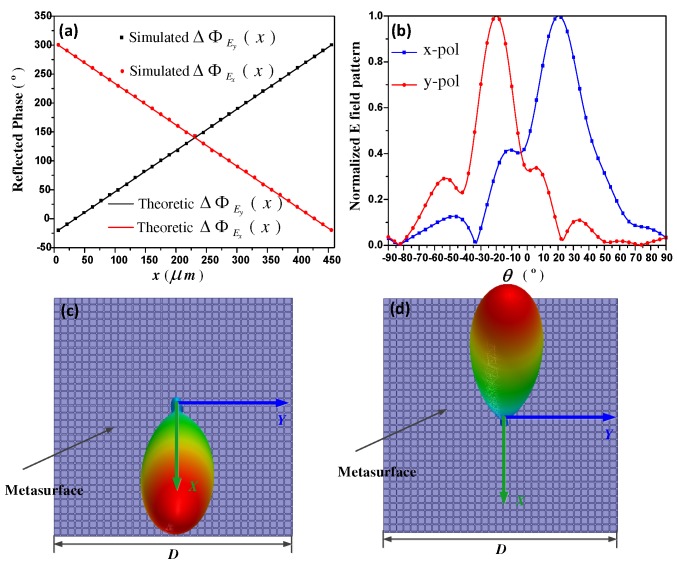
(**a**) The required phase distribution of function F2. $\Delta \Phi_{E_{x,y}}\left(x\right)$ represents the x- or y-polarization wave reflection phase distribution along the +x direction, which is an one-dimension (1D) form of $\Delta \Phi_{E_{x,y}}\left(x,y\right)$ when *y* keeps constant in Equation (2). Theoretic results are obtained from analytical calculation, and simulation results are obtained from numerical simulation, respectively; (**b**) is the 2-D scattering patterns of x- and y-polarized waves; (**c**) and (**d**) are the 3-D scattering patterns of x- and y-polarized waves, respectively.

Similar to F1, function F2 is designed to deflect the x- and y-waves to the directions of ($\varphi_{E_{x}}$ = 0${}^{\circ }$, $\theta_{E_{x}}$ = 20${}^{\circ }$) and ($\varphi_{E_{y}}$ = 180${}^{\circ }$, $\theta_{E_{y}}$ = −20${}^{\circ }$), respectively. In this case, due to only the deflection in the *x* direction needs to be considered, the required reflection phase distribution is merely varied in the *x* direction, as depicted in Figure 4a. Using the similar approach as in F1 designing, controlling parameters $\mu_{c1}$ and $\mu_{c2}$ in each unit-cell can be determined. Here, both of $\mu_{c1}$ and $\mu_{c2}$ is gradually changed with opposite trends along the +x direction. Simulated 2-D and 3-D scattering patterns are exhibited in Figure 4b–d. It is presented that an arbitrarily linear polarized incident wave is separated into the two x- and y-polarized beams, with expected directions of ($\varphi_{E_{x}}$ = 0${}^{\circ }$, $\theta_{E_{x}}$ = 20${}^{\circ }$) and ($\varphi_{E_{y}}$ = 180${}^{\circ }$, $\theta_{E_{y}}$ = −20${}^{\circ }$), respectively.

### 3.2. Graphene-Based Metasurface for Arbitrarily Linearly Polarized Beam Deflecting

**Figure 5:**
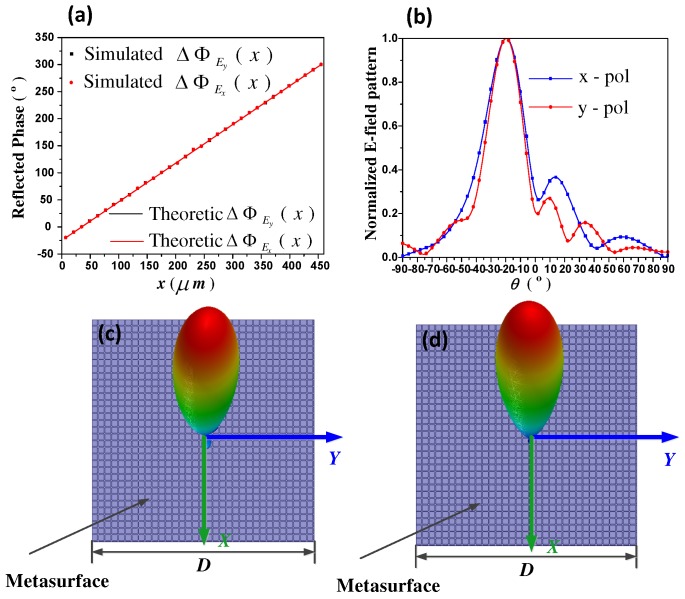
(**a**) The required phase distribution of function F3. $\Delta \Phi_{E_{x,y}}\left(x\right)$ represents the x- or y-polarization wave reflection phase distribution along the +x direction, which is an one-dimension (1D) form of $\Delta \Phi_{E_{x,y}}\left(x,y\right)$ when *y* keeps constant in Equation (2). Theoretic results are obtained from analytical calculation, and simulation results are obtained from numerical simulation, respectively. (**b**) is the 2-D scattering patterns of x- and y-polarized waves. (**c**) and (**d**) are the 3-D scattering patterns of x- and y-polarized waves, respectively.

Based on previous two functions, function F3 expected to deflect both of x- and y-polarized components of an incident linear wave to the same direction of ($\varphi_{E_{x}}$ = 180${}^{\circ }$, $\theta_{E_{x}}$ = −20${}^{\circ }$) and ($\varphi_{E_{y}}$ = 180${}^{\circ }$, $\theta_{E_{y}}$ = −20${}^{\circ }$). According to Equation (2), reflection phase distribution is theoretic calculated as in Figure 5a, where the required reflection phase of both of x- and y-polarized waves are gradually increased along the +x direction, with a constant phase gradient. We can determine the exact value of chemical potential in each unit-cell by the relationship of reflection phases and chemical potentials provided by Figure 1d. Furthermore, Figure 5a presents 33 discrete reflection phases extracted from Figure 1d according to the phase distribution requirement. In this case, both of $\mu_{c1}$ and $\mu_{c2}$ are gradually changing along the +x direction. Figure 5b–d demonstrate both of the 2-D and 3-D scattering patterns of x- and y-polarized waves. Obviously, it is clearly demonstrated that both of the x- and y-polarized waves are deflected to the same directions of ($\varphi_{E_{x}}$ = 180${}^{\circ }$, $\theta_{E_{x}}$ = −20${}^{\circ }$) and ($\varphi_{E_{y}}$ = 180${}^{\circ }$, $\theta_{E_{y}}$ = −20${}^{\circ }$). It means that a graphene-based metasurface which can deflect arbitrarily linear polarized incident wave to a specific direction is realized.

### 3.3. Graphene-Based Metasurface for Linearly-to-Circularly Polarized Converting

**Figure 6:**
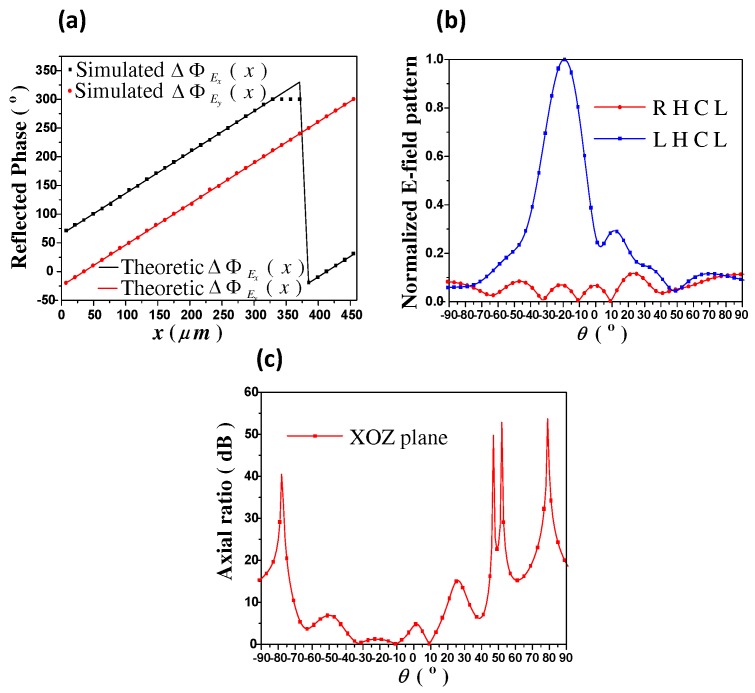
(**a**) The required phase distribution of function F4. $\Delta \Phi_{E_{x,y}}\left(x\right)$ represents the x- or y-polarization wave reflection phase distribution along the +x direction, which is an one-dimension (1D) form of $\Delta \Phi_{E_{x,y}}\left(x,y\right)$ when *y* keeps constant in Equation (2). Theoretic results are obtained from analytical calculation, and simulation results are obtained from numerical simulation, respectively; (**b**) The 2-D scattering patterns of both of the right hand circularly polarization (RHCP) and left hand circularly polarization (LHCP) components; (**c**) The axial ratio of the reflected wave.

Finally, F4 is designed to perform as a linear-to-circular polarization converter, which can deflect both x- and y-polarized reflected waves to the same direction of (180${}^{\circ }$, −20${}^{\circ }$), with a phase difference of 90${}^{\circ }$. In this case, the metasurface only requires *x* direction phase distributions, as exhibited in Figure 6a. Therefore, chemical potentials $\mu_{c1}$ and $\mu_{c2}$ can be obtained accordingly. It is worth noting here that because there is only a reflection phase span of 320${}^{\circ }$, some unit-cells on the metasurface cannot obtain the theoretic values. Therefore, we have to use approximate values in these locations. Due to there are only a few such approximate points, the total scattering performances have not been affected. Figure 6b illustrates the 2-D scattering patterns of both of the right hand circularly polarization (RHCP) and left hand circularly polarization (LHCP) components. Obviously, RHCP wave has been deflected to the expected direction of (180${}^{\circ }$, −20${}^{\circ }$). Moreover, the axial ratio of the reflected wave is given in Figure 6c, demonstrating excellent circularly-polarization in the desired direction. Hence, by proper chemical potential tuning, a linear-to-circular converter is proposed.

At the end of this section, it is worth noting that, inspired by practical applications such as telecommunication [37], bio-sensing [38], antennas [39] and automotive [40], our work provided a soft-ware driven reconfigurability, and can pave the way to higher integration for these applications.

## 4. Conclusions

In conclusion, we have introduced a reconfigurable multi-functional graphene-based metasurface with a high degree of miniaturization and integration. By virtue of the reconfigurability of reflection patterns, independent tailoring of the orthogonal linearly polarized terahertz wave is realized. The graphene-based unit-cell consists of two orthogonal graphene strips and a grounded substrate, which has anisotropic responses for each of orthogonal polarizations (x-polarized and y-polarized waves). The reflection phases of both x- and y-polarized waves can be controlled independently through separate electrical tuning. Based on the proposed metasurface, functionalities including beam splitting, beam deflection, and linear-to-circular polarization converting using a shared aperture are numerically demonstrated and analyzed. Simulation results demonstrate excellent performance, which is consistent with the theorized expectations. It is worth emphasizing here that, taking into account of the practical fabrication, we can easily fabricate the graphene-based unit-cell which consists of 5 layers, including a graphene-strip layer, an alumina layer, a polysilicon layer, a quartz glasses layer, and a ground plate. The polysilicon can be utilized as an electrode. The surface complex impedance of graphene-patch can be dynamically controlled by varying the DC voltage (VDC) between the graphene-patch and the polysilicon. Concrete procedure to fabricate these kinds of graphene-based metasurface can be found in Ref. [41], supporting the feasibility of our work. Therefore, our proposed graphene-based metasurface can be utilized to realize extremely light-weight, ultra-compact, multi-functional, and reconfigurable electromagnetic structures for various terahertz applications. In addition, we must state that this work has only dynamically realized some expected functions. And as a devices, the performances of the proposed metasurface still need to be optimized. Therefore, future works may be focused on performance optimization and experimental verification. This work paves the way for enhancing the miniaturization of modern electronic/optical devices and potentially has important applications in the next-generation information systems for communication, sensing, and imaging.
